# Mesenchymal Stem Cells May Alleviate the Intervertebral Disc Degeneration by Reducing the Oxidative Stress in Nucleus Pulposus Cells

**DOI:** 10.1155/2022/6082377

**Published:** 2022-10-03

**Authors:** Yongzhao Zhao, Qian Xiang, Yunzhong Cheng, Jialiang Lin, Shuai Jiang, Weishi Li

**Affiliations:** ^1^Department of Orthopaedics, Peking University Third Hospital, Beijing, China; ^2^Beijing Key Laboratory of Spinal Disease Research, Beijing, China; ^3^Engineering Research Center of Bone and Joint Precision Medicine, Ministry of Education, Beijing, China; ^4^Department of Orthopedic Surgery, Beijing Chao-Yang Hospital, Capital Medical University, Beijing, China

## Abstract

**Background:**

Stem cell therapy is a promising therapeutic modality for intervertebral disc degeneration (IDD). Oxidative stress is a vital contributor to the IDD; however, the definite role of oxidative stress in stem cell therapy for IDD remains obscure. The aim of this study was to determine the vital role of oxidative stress-related differentially expressed genes (OSRDEGs) in degenerative NPCs cocultured with mesenchymal stem cells (MSCs).

**Methods:**

A series of bioinformatic methods were used to calculate the oxidative stress score and autophagy score, identify the OSRDEGs, conduct the function enrichment analysis and protein-protein interaction (PPI) analysis, build the relevant competing endogenous RNA (ceRNA) regulatory networks, and explore the potential association between oxidative stress and autophagy in degenerative NPCs cocultured with MSCs.

**Results:**

There was a significantly different oxidative stress score between NPC/MSC samples and NPC samples (*p* < 0.05). Forty-one OSRDEGs were selected for the function enrichment and PPI analyses. Ten hub OSRDEGs were obtained according to the PPI score, including JUN, CAT, PTGS2, TLR4, FOS, APOE, EDN1, TXNRD1, LRRK2, and KLF2. The ceRNA regulatory network, which contained 17 DElncRNAs, 240 miRNAs, and 10 hub OSRDEGs, was constructed. Moreover, a significant relationship between the oxidative stress score and autophagy score was observed (*p* < 0.05), and 125 significantly related gene pairs were obtained (|*r*| > 0.90, *p* < 0.05).

**Conclusion:**

Stem cell therapy might repair the degenerative IVD via reducing the oxidative stress through the ceRNA regulatory work and restoration of autophagy in degenerative NPCs. This research could provide new insights into the mechanism research of stem cell therapy for IDD and potential therapeutic targets in the IDD treatment.

## 1. Background

Low back pain (LBP) has become a very common health concern in the modern society, which generates a social and economic burden to human beings [1–3]. It is estimated that approximately 80% of population experience the LBP at least once in their lifetimes [4]. Intervertebral disc degeneration (IDD) is the principal contributor to the LBP [2, 5]. IDD is an inflammatory-catabolic process triggered by a series of pathogenic factors, including gene susceptibility, increased mechanical stress, abnormal immunity, metabolic disorders, and oxidative stress [3, 6, 7]. The standard treatments for LBP caused by IDD include the bed rest, administration of nonsteroid anti-inflammatory drugs and analgesics, discectomy, and lumbar interbody fusion [2, 5]. However, the existing treatments can only relieve the clinical symptoms instead of reversing the degeneration process. Therefore, novel therapies targeting the degeneration process are urgently needed.

In recent years, stem cell therapy has shown a promising effect and potential clinical applicability in the management of IDD [8–12]. Accumulating evidence has indicated that mesenchymal stem cells (MSCs) might exert therapeutic functions mostly through the paracrine process, such as the release of growth factors, cytokines, extracellular vesicles, and noncoding RNAs [8]. However, the definite underlying mechanisms remain unclear. Oxidative stress has been demonstrated to play important roles in the development of IDD [13, 14]. Under normal circumstances, the microenvironment of intervertebral disc (IVD) tissue is hypoxic, and there is a dynamic balance between the generation and scavenging of intracellular reactive oxide species [15]. However, the oxidative stress occurs when this balance is disrupted, which can lead to senescence and apoptosis of nucleus pulposus cells (NPCs), and degradation of extracellular matrix [16].

Long noncoding RNAs (lncRNAs) refer to a type of noncoding RNA longer than 200 nucleotides [17, 18]. Although lncRNA lacks the ability to encode proteins, lncRNAs can act as the competing endogenous RNAs (ceRNAs) by sponging the microRNAs (miRNAs) to repress the translation of genes [19, 20]. Autophagy is a well-known conserved cellular process through which cells can realize the self-protection by scavenging the unwanted senescent organelles and misfolded proteins [21, 22]. The dysregulation of autophagy has been proved to associate with the development of several human diseases, including the IDD [21, 23]. Previous studies have shown that the oxidative stress could be relieved by activating the autophagy in degenerative NPCs, thereby reducing the apoptosis and degradation of extracellular matrix [22, 24, 25]. Nevertheless, to our knowledge, few articles focus on the effects of MSCs on the alleviation of oxidative stress via regulating the autophagy in IDD.

With the huge improvement of sequencing techniques, many key genes and noncoding RNAs associated with the IDD have been determined using the bioinformatic approaches [13, 26, 27]. We previously reported that oxidative stress is an important pathogenic factor for IDD [13]. Wang et al. found that infiltrating macrophages play important roles in the pathogenesis of IDD [26]. In Li et al. study, 305 genes closely related to IDD were obtained, and the authors also reported that DNA repair, oxidative phosphorylation, peroxisome, IL-6-JAK-STAT3 signaling, and apoptosis contributed to the development of IDD [27]. However, few bioinformatic analysis focusing on the role of stem cell therapy in the management of IDD are published to date. Hence, this study was conducted to explore the underlying mechanisms of stem cell therapy in the management of IDD using the strict and mature bioinformatic algorithms based on the relevant sequencing data.

## 2. Materials and Methods

**Figure 1:**
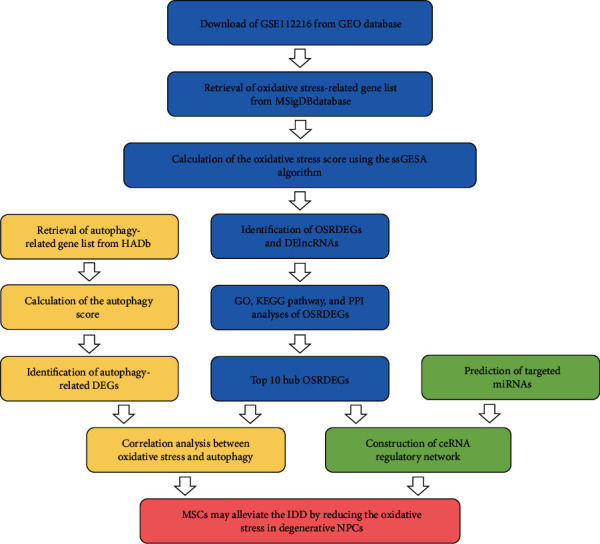
Flow chart of the bioinformatic analysis in the study.

This study has been approved by the Ethics Committee of Peking University Third Hospital, and the informed consent was not necessary because all data was obtained from public databases. The flow chart of this study has been shown in Figure 1.

### 2.1. Data Collection and Processing

Gene expression data of mRNAs and lncRNAs in GSE112216 was downloaded from Gene Expression Omnibus (GEO) database (https://www.ncbi.nlm.nih.gov/geo/). This dataset contained the gene chip sequencing data of 3 NPC/MSC samples and 3 NPC samples, and compare the mRNA and lncRNA expression of degenerative NPCs cocultured with adipose-derived MSCs with degenerative NPCs solely. The oxidative stress-related gene (OSRG) list was extracted from the Gene Set: GOBP_RESPONSE_TO_OXIDATIVE_STRESS in Molecular Signatures Database (http://www.gsea-msigdb.org/gsea/msigdb/index.jsp) [26] (Supplementary Table 1). Besides, the autophagy-related gene list was obtained from the Human Autophagy Database (http://www.autophagy.lu/index.html) (Supplementary Table 2).

### 2.2. Determination the Alteration of OSRGs during the Coculture Process between NPCs and MSCs

The single sample gene set enrichment analysis (ssGSEA) is a bioinformatic approach to determine that whether a priori defined set of genes has statistical significance and concordant differences between two biological conditions for a single sample [13]. To investigate the alteration of OSRGs during the coculture process between NPCs and MSCs, the ssGSEA algorithm was applied to calculate the oxidative stress score of each cell sample [28]. The oxidative stress score was compared between NPC/MSC samples and NPC sample.

### 2.3. Identification of Differentially Expressed Genes (DEGs), Oxidative Stress-Related DEGs (OSRDEGs), and Differentially Expressed lncRNAs (DElncRNAs)

Both DEGs and DElncRNAs were obtained from the GSE112216 with the criterion of adjust *p* < 0.05 and fold change > 1.50. The OSRDEGs were obtained with the intersection of DEGs and OSRGs using the Venn diagram. Volcano plots and heat maps were generated using the R package ggplot2.

### 2.4. Functional Enrichment Analysis and Protein-Protein Interaction (PPI) Analysis of OSRDEGs

Gene ontology (GO) analysis was conducted to explore the enriched biological process, cell component, and molecular function of OSRDEGs. Besides, the related signaling pathways of OSRDEGs were determined using the Kyoto Encyclopedia of Genes and Genomes (KEGG) analysis. The GO and KEGG functional enrichment analyses were performed using the DAVID database (https://david.ncifcrf.gov/) [29]. GO and KEGG items with *p* < 0.05 were considered as significantly enriched, and some of significantly enrich items were visualized using the R package ggplot2. The PPI analysis was conducted using the STRING database (https://cn.string-db.org/), and protein pairs with score > 0.40 were further used to build the PPI network using the Cytoscape software (https://cytoscape.org/). The PPI score was calculated using the Degree method in the cytoHubba plug-in, and top 10 OSRDEGs ranked by the PPI score were considered as the hub OSRDEGs.

### 2.5. Construction of DElncRNA-miRNA-Hub OSRDEG Regulatory Network

The correlation analysis between DElncRNAs and hub OSRDEGs was performed, and DElncRNA-OSRDEG pairs with *r* > 0.95 and *p* < 0.05 were selected. The targeted miRNAs for 10 hub OSRDEGs were predicted using TargetScan database (http://www.targetscan.org/vert_80/) [30]. The targeted miRNAs for DElncRNAs were predicted using the ENCORI database (https://starbase.sysu.edu.cn/) [28]. Ultimately, the DElncRNA-miRNA-hub OSRDEG regulatory network was constructed using the Cytoscape software.

### 2.6. Correlation Analysis between Oxidative Stress and Autophagy

To further explore the potential role of autophagy during the coculture process, the autophagy score for each cell sample was calculated using the ssGSEA algorithm [5], and compared between NPC/MSC samples and NPC samples. To obtain the autophagy-related DEGs, the intersection between autophagy-related genes and DEGs was conducted using the Venn diagram. To detect the potential relationship between oxidative stress and autophagy in degenerative NPCs cocultured with MSCs, the correlation analysis between oxidative stress score and autophagy score was conducted. Furthermore, the relationship between hub OSRDEGs and autophagy-related DEGs was explored using the correlation analysis.

### 2.7. Statistical Analysis

All statistical analyses were performed using the R software 4.1.2. The ssGSEA score for oxidative stress and autophagy between NPC/MSC samples and NPC samples were compared using the Student's *t*-test, and *p* < 0.05 indicated there was a significant difference between NPC/MSC samples and NPC samples. Correlation analysis was conducted using the Pearson test. All *p* values were two sides, and *p* value less than 0.05 indicated there was a significant difference.

## 3. Results

### 3.1. MSCs Might Alleviate the Oxidative Stress in Degenerative NPCs

**Figure 2:**
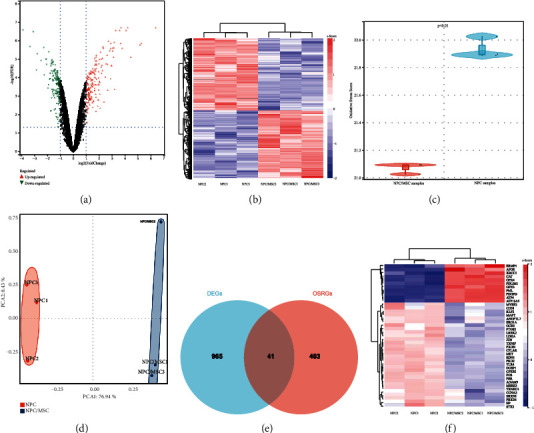
Determination of OSRDEGs in degenerative NPCs. (a) Volcano plot of DEGs; (b) Heat map of DEGs; (c) Comparison of oxidative stress score between NPC/MSC samples and NPC samples; (d) Principal component analysis of OSRGs; (e) Venn diagram to obtain the OSRDEGs; (f) Heat map of OSRDEGs.

As shown in Figure 2(a), according to the preset criterion (fold change > 1.5, *p* < 0.05), a total of 106 DEGs were determined, and the clustering analysis showed these DEGs could clearly distinguish the NPC/MSC samples and NPC samples (Figure 2(b)). Oxidative stress is an important contributor to the IDD [6, 13]. To explore whether MSCs changed the oxidative stress status of degenerative NPCs, the oxidative stress score for each cell sample was calculated. There was a significant difference between NPC/MSC samples and NPC samples in terms of oxidative stress score (Figure 2(c)). The principal component analysis showed OSRGs could clearly distinguish the NPC/MSC samples and NPC samples (Figure 2(d)), which indicated that stem cell therapy might treat the IDD via relieving the oxidative stress in NPCs. To further investigate the underlying mechanisms, forty-one OSRDEGs were obtained by intersecting the DEGs with OSRGs (Figure 2(e)), and 11 of them were upregulated and 30 of them were downregulated (Table 1). As shown in the heat map (Figure 2(f)), the OSRDEGs significantly differed between the NPC/MSC samples and NPC samples.

### 3.2. Function Enrichment Analysis and PPI Analysis of OSRDEGs

**Figure 3:**
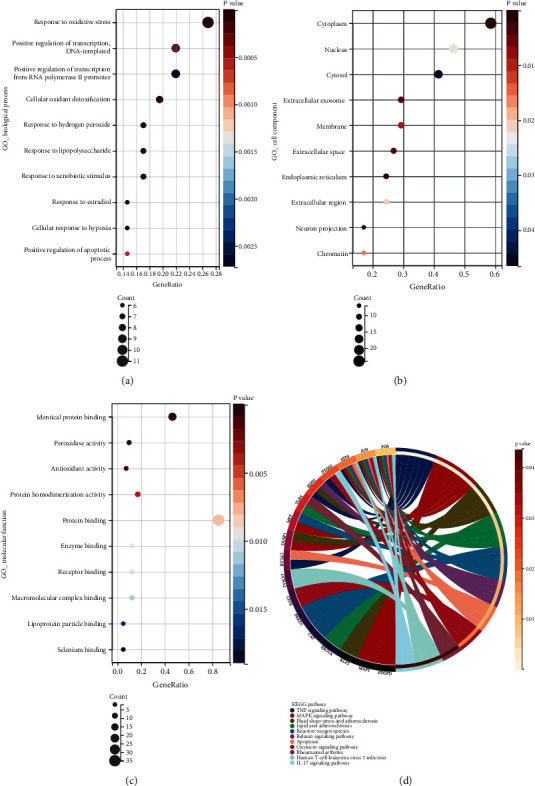
Functional enrichment analysis of OSRDEGs. (a) GO_ biological process; (b) GO _ cell component; (c) GO_ molecular function; (d) KEGG analysis.

The identified OSRDEGs were mapped into the GO term and KEGG pathway enrichment analyses. As shown in Figure 3(a), the following biological processes were significantly affected: Response to oxidative stress, positive regulation of transcription and DNA-templated, positive regulation of transcription from RNA polymerase II promoter, cellular oxidant detoxification, and so on. The most enriched cellular component terms were Cytoplasm, Nucleus, Cytosol, Extracellular exosome, and so on (Figure 3(b)). The most enriched molecular function terms included Identical protein binding, Peroxidase activity, Antioxidant activity, Protein homodimerization activity, and so on (Figure 3(c)). With respect to the KEGG pathway enrichment analysis, the following pathways were most affected: TNF signaling pathway, MAPK signaling pathway, Reactive oxygen species, Apoptosis, IL-17 signaling pathway, and so on (Figure 3(d)).

**Figure 4:**
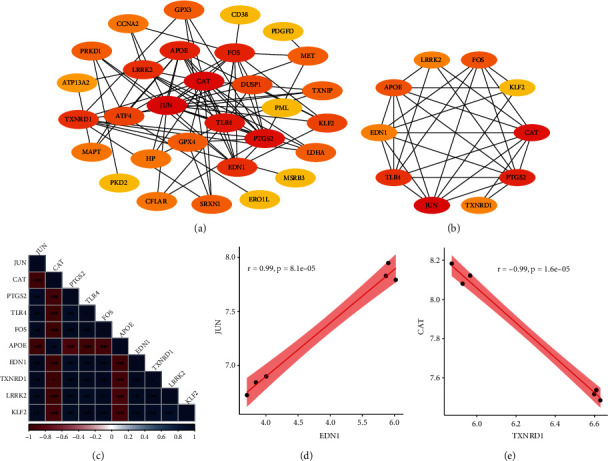
PPI analysis of OSRDEGs. (a) PPI analysis; (b) Top 10 hub OSRDEGs; (c) Correlation analysis among 10 hub OSRDEGs; (d) Correlation analysis between JUN and EDN1; (e) Correlation analysis between CAT and TXNRD1.

The PPI analysis was conducted using the STRING database and visualized using the Cytoscape software (Figure 4(a)). Top 10 hub genes were obtained according to the PPI score, including JUN, CAT, PTGS2, TLR4, FOS, APOE, EDN1, TXNRD1, LRRK2, and KLF2 (Figure 4(b)). As listed in Table 2, 8 hub OSRDEGs were downregulated and 2 hub OSRDEGs were upregulated in NPC/MSC samples when compared to NPC samples. Moreover, the correlation analysis among these 10 hub OSRDEGs was conducted, and 45 significantly related pairs (|*r*| > 0.90, *p* < 0.05) were observed (Figure 4(c)). JUN-EDN1 was the most positively related pair (*r* = 0.99, *p* < 0.01) (Figure 4(d)), and CAT-TXNRD1 was the most negatively related pair (*r* = −0.99, *p* < 0.05) (Figure 4(e)).

### 3.3. Construction of DElncRNA-miRNA-Hub OSRDEGs Regulatory Network

**Figure 5:**
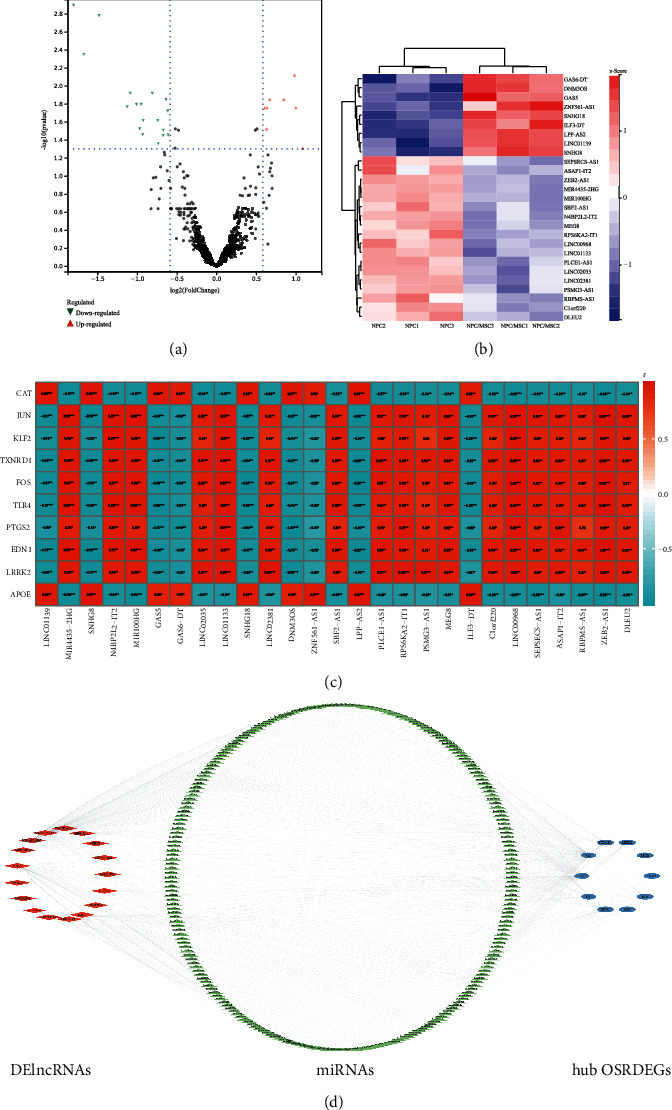
Construction of ceRNA regulatory network. (a) Volcano plot of DElncRNAs; (b) Heat map of DElncRNAs; (c) Correlation analysis between hub OSRDEGs and DElncRNAs; (d) The ceRNA regulatory network containing 17 DElncRNAs, 240 miRNAs, and 10 hub OSRDEGs.

LncRNAs can exert the important biological functions as the miRNA sponges in the ceRNA regulatory network [29, 31]. A total of 27 DElncRNAs were determined (fold change > 1.50, *p* < 0.05) (Figure 5(a)), and the heat map showed these DElncRNAs could obviously distinguish the NPC/MSC samples and NPC samples (Figure 5(b)). To construct the ceRNA regulatory work, the correlation analysis between DElncRNAs and hub OSRDEGs was conducted, and DElncRNA-OSRDEG pairs with *r* > 0.95 and *p* < 0.05 were selected to construct the ceRNA regulatory network (Figure 5(c)). The targeted miRNAs for DElncRNA-OSRDEG pairs were predicted using the ENCORI database and TargetScan database. Ultimately, a total of 17 DElncRNAs, 240 miRNAs, and 10 hub OSRDEGs were applied to construct the ceRNA regulatory network (Figure 5(d)) (Supplementary Table 3).

### 3.4. Relationship between Hub OSRDEGs and Autophagy-Related DEGs

**Figure 6:**
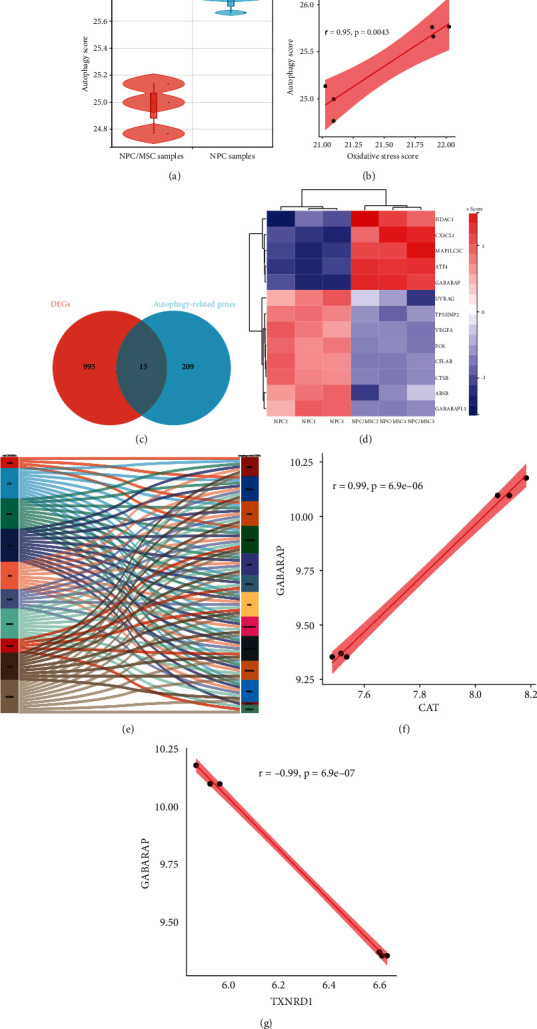
Correlation analysis between oxidative stress and autophagy in degenerative NPCs. (a) Comparison of autophagy score between NPC/MSC samples and NPC samples; (b) Correlation analysis between oxidative stress score and autophagy score; (c) Venn diagram to obtain the autophagy-related DEGs; (d) Heat map of autophagy-related DEGs; (e) Correlation analysis between top 10 hub OSRDEGs and 13 autophagy-related DEGs; (f) Correlation analysis between GABARAP and CAT; (g) Correlation analysis between GABARAP and TXNRD1.

Previous studies have shown that autophagy played a protective role against the oxidative stress in degenerative NPCs [24, 30, 32]. In this research, a significantly different autophagy score between NPC/MSC samples and NPC samples was observed (Figure 6(a)). And there was an obvious association between oxidative stress score and autophagy score (Figure 6(b)), which indicated that MSCs might resist again the oxidative stress through restoring the autophagy in degenerative NPCs. To further explore the underlying mechanisms, thirteen autophagy-related DEGs were obtained through the intersection between DEGs and autophagy-related genes (Figure 6(c)), and the cluster analysis showed these autophagy-related DEGs could distinctly distinguish the NPC/MSC samples and NPC samples (Figure 6(d)). The correlation analysis between hub OSRDEGs and autophagy-related DEGs was conducted, and 125 significantly related pairs were obtained (|*r*| > 0.90, *p* < 0.05) (Figure 6(e)). GABARAP-CAT was the most positively related pair (*r* = 0.99, *p* < 0.01) (Figure 6(f)) and GABARAP-TXNRD1 was the most negatively related pair (*r* = −0.99, *p* < 0.01) (Figure 6(g)).

## 4. Discussion

IDD has become the principal contributor to the LBP, which heavily affects the life quality of patients and brings a huge economic burden to the society [2, 5]. Stem cell therapy has been considered as a promising therapeutic option for IDD, however, the involved underlying mechanisms remain unclear to date [33–36]. In the current study, we used a series of strict bioinformatic algorithms based on the sequencing data to determine the potential mechanisms involved in the stem cell therapy for IDD. We observed a significantly different oxidative stress score between NPC/MSC samples and NPC samples, which indicated that MSCs might alleviate the IDD via suppressing the oxidative stress in degenerative NPCs. Then, we determine the OSRDEGs, and explored the potential biological process and signaling pathways relevant to these OSRDEGs. Moreover, we got 10 hub OSRDEGs most worthwhile further exploring, and constructed the ceRNA regulatory network. More importantly, we found that autophagy might play an important role in the process of MSCs relieving the oxidative stress in degenerative NPCs. To the best knowledge of us, this study was the first bioinformatic analysis to investigate the possible mechanisms involved in the stem cell therapy for IDD.

Oxidative stress has been demonstrated to play a key role in the pathogenesis of IDD [6, 13, 16]. Oxidative stress could induce the apoptosis of normal NPCs, destroy the matrix proteins, and thus damage the mechanical characteristics of IVDs [16]. Some studies have explored the potential role of oxidative stress in the stem cell therapy for IDD [33, 37] . Hu et al. study showed that bone MSCs could alleviate the compression-induced apoptosis of NPCs through inhibiting the oxidative stress via the exosomes [37]. Similarly, Chen et al. reported that bone MSCs could relieve the compression-induced mitochondrial damage of NPCs through reducing the reactive oxygen species level and maintaining the mitochondrial functions [33]. In the current study, we observed a significantly different oxidative stress score between NPC/MSC samples and NPC samples, which indicated that stem cell therapy might improve the IDD through alleviating the oxidative stress in degenerative NPCs.

To further explore the potential underlying mechanisms involved in the stem cell therapy for IDD, we obtained 41 OSRDEGs and explored their main biological functions. The most enriched biological process was Response to oxidative stress, cellular component was Cytoplasm, and molecular function was Identical protein binding. More importantly, we also investigated the potential signaling pathways involved in the repair process of NPCs cocultured with MSCs, and some of these signaling pathways have been proved to play important roles in the pathophysiology of IDD [38–42]. TNF signaling pathway and IL-17 signaling pathway both were inflammation-related pathways, which indicated that MSCs might reduce the oxidative stress, and then improve the inflammatory status of degenerative NPCs [38, 39]. MAPK signaling pathway was another vital biological pathway in the development of IDD [40–42]. Zhang et al. reported that platelet-derived growth factor-BB could prevent the IDD through activating the MAPK signaling pathway [40]. Cui et al. study showed that microRNA-129-5p could alleviate the IDD via blocking the LRG1-mediated p38 MAPK activation [41]. Sun et al. research indicated that calcitonin gene-related peptide could regulate the apoptosis and inflammation of NPCs via the MAPK signaling pathway during the IDD [42]. For the first time, we discovered that Relaxin signaling pathway and Oxytocin signaling pathway might exert vital functions in the biological remediation of degenerative NPCs cocultured with MSCs. Both relaxin and oxytocin have been demonstrated to exert important protective effects in human diseases by inhibiting the cell apoptosis [43–47]. Therefore, we speculate that MSCs may relieve the oxidative stress by activing the Relaxin or Oxytocin signaling pathways, and then prevent the apoptosis of NPCs, which is very worthy of further investigation.

Through a series of bioinformatic methods, 10 hub OSRDEGs were selected, including JUN, CAT, PTGS2, TLR4, FOS, APOE, EDN1, TXNRD1, LRRK2, and KLF2. PTGS2 was upregulated in degenerative NPCs, and associated with the inflammation in IDD [48]. TLR4 inhibition could reduce the LBP, pain-related neuroplasticity, and inflammation of disc in mice [49]. Knockout of APOE could accumulate the selective inflammatory catabolic factors, which aggravated the imbalances between catabolic and anabolic factors and deteriorated the premature IDD [50]. LRRK2 contributed to the pathogenesis of IDD, and knockdown of LRRK2 could inhibit the oxidative stress induced apoptosis through the mitophagy [51]. The potential roles of JUN, CAT, FOS, EDN1, TXNRD1, and KLF2 in IDD have not been investigated in details up to now, and deserve the further investigation. Plenty of studies have shown that lncRNAs could sponge miRNAs, also named as ceRNA regulatory network, to regulate the gene expression at a posttranscriptional level [52, 53]. To further explore the potential underlying mechanisms associated with hub OSRDEGs, we constructed the DElncRNA-miRNA- hub OSRDEG regulatory network containing 17 DElncRNAs, 240 miRNAs, and 10 hub OSRDEGs, which should be further studied in the future.

Autophagy is a catabolic process that recycles the cellular components and damaged organelles caused by various stress status [16, 54]. The autophagy level was higher in degenerative NPCs compared with normal NPCs, which indicated that autophagy might be involved in the deterioration of IDD [55]. Many investigations have indicated that autophagy was an important protective factor for IVD, and the restoration of autophagy was a promising research direction in IDD [13, 56–58]. Some studies have indicated that MSCs could significantly increase the autophagy level, and reduce the apoptosis of NPCs [25, 59]. More importantly, there was a close relationship between oxidative stress and autophagy in IDD. Chen et al. found that the overproduction of reactive oxygen species could enhance the autophagy via the AMPK/mTOR pathway in rat NPCs [60]. Moreover, Park et al. found that high glucose-induced oxidative stress could improve the autophagy by mitochondrial damage in rat notochordal cells [61]. Chen et al. reported that H_2_O_2_ could stimulate an early autophagy response through the ERK/m-TOR signaling pathway [62]. In the current study, we obtained 13 autophagy-related DEGs and performed the correlation analysis between hub OSRDEGs and autophagy-related DEGs. At last, 125 significantly related pairs were obtained, which showed that autophagy might expert vital functions in the stem cell therapy for IDD. The GABARAP-CAT pair was the most positively related pair and GABARAP-TXNRD1 pair was the most negatively related pair, and both of them should be firstly investigated in the future.

There were some limitations in the current study. First, this study was conducted based on the analysis of sequencing data. Therefore, our findings need further in vivo or vitro experiment validation. Second, oxidative stress was only one of the important pathogenic factors for IDD, and stem cell therapy might also repair the degenerative IVD though other pathways, such as relieving the inflammation. Third, the sequencing data used in this study was obtained from the cell samples, which could not completely simulate the degenerative IVDs treated with stem cell therapy. Forth, only six sequencing cell samples from one GEO dataset were used in this study, which might reduce the reliability of findings. Fifth, the MSCs used in this study were extracted from adipose tissues, however, there were several other sources for MSCs, such as bone marrows and embryonal tissues, which needed further investigation. Despite these limitations, the current study, for the first time, indicated that stem cell therapy might repair the degenerative IVD through resisting the oxidative stress via the ceRNA regulatory network and restoration of autophagy in degenerative NPCs.

## 5. Conclusion

Stem cell therapy might repair the degenerative IVD via reducing the oxidative stress through the ceRNA regulatory work and restoration of autophagy in degenerative NPCs. Further experiment studies should be conducted to validate our findings in the future.

## Figures and Tables

**Table 1:** Detailed information of 41 OSRDEGs.

Expression	Gene symbols	Number (*n*)
Upregulated	ERMP1, CAT, ATF4, GPX3, PML, GPX4, XRCC1, APOE, ATP13A2, PDLIM1, and PDGFD	11
Downregulated	EDN1, FOS, PKD2, KLF2, DUSP1, MET, GCH1, PTGS2, LRRK2, JUN, LDHA, TXNIP, FER, MSRB3, TLR4, CFLAR, MYEF2, CD38, ADAM9, CPEB2, SRXN1, MAPT, PXDN, PRKD1, HP, TXNRD1, ANGPTL7, CCNA2, STX2, and ERO1A	30

OSRDEGs, oxidative stress related differentially expressed genes.

**Table 2:** Detailed information of top 10 hub OSRDEGs.

Gene symbols	Full names	Gene function	Log2 (fold change)	*P* value	Regulation
JUN	Jun proto-oncogene	This gene encodes a protein which can regulate the gene expression via interacting directly with specific target DNA sequences.	-1.03	<0.01	Down
CAT	Catalase	This gene encodes the catalase, which is an important antioxidant enzyme in the bodies against the oxidative stress.	0.62	<0.01	Up
PTGS2	Prostaglandin-endoperoxide synthase 2	The protein encoded by this gene is a vital enzyme in the process of prostaglandin biosynthesis, and acts both as a dioxygenase and as a peroxidase.	-1.12	<0.01	Down
TLR4	Toll like receptor 4	The protein encoded by this gene is a member of the toll-like receptor family, which is involved in the pathogen recognition and activation of inherent immunity.	-0.88	<0.01	Down
FOS	Fos proto-oncogene	This gene encodes one member of leucine zipper proteins that can dimerize with proteins of the JUN family to form the transcription factor complex AP-1.	-1.63	<0.01	Down
APOE	Apolipoprotein E	The protein encoded by this gene is a major apoprotein of the chylomicron, which is indispensable for the catabolism of triglyceride-rich lipoprotein constituents.	1.19	<0.01	Up
EDN1	Endothelin 1	This gene encodes a preproprotein that is proteolytically processed to produce a secreted peptide. This gene is involved with the tumorigenesis and pulmonary arterial hypertension.	-2.08	<0.01	Down
TXNRD1	Thioredoxin reductase 1	The protein encoded by this gene is a member of the pyridine nucleotide-disulfide oxidoreductase family, and the thioredoxin system.	-0.69	<0.01	Down
LRRK2	Leucine rich repeat kinase 2	This gene is a member of the leucine-rich repeat kinase family, and the dysregulated expression of this gene may lead to the Parkinson disease-8.	-1.09	<0.01	Down
KLF2	Kruppel like factor 2	The protein encoded by this gene is a member of Kruppel family of transcription factors. It plays an important role in the adipogenesis, embryonic erythropoiesis, epithelial integrity, inflammation and t-cell viability.	-1.29	<0.01	Down

OSRDEGs, oxidative stress-related differentially expressed genes.

## Data Availability

The original contributions presented in the study are included in the article/supplementary material; further inquiries can be directed to the corresponding author/s.

## Abbreviations

LBP: Low back pain
IDD: Intervertebral disc degeneration
MSCs: Mesenchymal stem cells
IVD: Intervertebral disc
NPCs: Nucleus pulposus cells
lncRNAs: Long noncoding RNAs
ceRNAs: Competing endogenous RNAs
miRNAs: microRNAs
GEO: Gene expression omnibus
OSRG: Oxidative stress-related gene
ssGSEA: Single sample gene set enrichment analysis
DEGs: Differentially expressed genes
OSRDEGs: Oxidative stress-related differentially expressed genes
DElncRNAs: Differentially expressed long noncoding RNAs
PPI: Protein-protein interaction
GO: Gene ontology
KEGG: Kyoto encyclopedia of genes and genomes.

## Supplementary Materials

Supplementary 1 Supplementary Table 1. The list of oxidative stress-related genes.

Supplementary 2 Supplementary Table 2 The list of autophagy-related genes.

Supplementary 3 Supplementary Table 3. Detailed information of ceRNA regulatory network.
