# Supplementary material for: Mesenchymal Stem Cells May Alleviate the Intervertebral Disc Degeneration by Reducing the Oxidative Stress in Nucleus Pulposus Cells
Source: Stem Cells Int. 2022 Oct 3;2022:6082377. doi: 10.1155/2022/6082377 (PMC9551678; doi:10.1155/2022/6082377)
Supplement: Supplementary 2 — Supplementary Table 2 The list of autophagy-related genes. [file 6082377.f2.docx]

| **Supplementary Table 2 The list of autophagy-related genes** |
| --- |
| **Gene symbol** |
| AMBRA1 |
| APOL1 |
| ARNT |
| ARSA |
| ARSB |
| ATF4 |
| ATF6 |
| ATG10 |
| ATG12 |
| ATG16L1 |
| ATG16L2 |
| ATG2A |
| ATG2B |
| ATG3 |
| ATG4A |
| ATG4B |
| ATG4C |
| ATG4D |
| ATG5 |
| ATG7 |
| ATG9A |
| ATG9B |
| ATIC |
| BAG1 |
| BAG3 |
| BAK1 |
| BAX |
| BCL2 |
| BCL2L1 |
| BECN1 |
| BID |
| BIRC5 |
| BIRC6 |
| BNIP1 |
| BNIP3 |
| BNIP3L |
| C12orf44 |
| C17orf88 |
| CALCOCO2 |
| CAMKK2 |
| CANX |
| CAPN1 |
| CAPN10 |
| CAPN2 |
| CAPNS1 |
| CASP1 |
| CASP3 |
| CASP4 |
| CASP8 |
| CCL2 |
| CCR2 |
| CD46 |
| CDKN1A |
| CDKN1B |
| CDKN2A |
| CFLAR |
| CHMP2B |
| CHMP4B |
| CLN3 |
| CTSB |
| CTSD |
| CTSL1 |
| CX3CL1 |
| CXCR4 |
| DAPK1 |
| DAPK2 |
| DDIT3 |
| DIRAS3 |
| DLC1 |
| DNAJB1 |
| DNAJB9 |
| DRAM1 |
| EDEM1 |
| EEF2 |
| EEF2K |
| EGFR |
| EIF2AK2 |
| EIF2AK3 |
| EIF2S1 |
| EIF4EBP1 |
| EIF4G1 |
| ERBB2 |
| ERN1 |
| ERO1L |
| FADD |
| FAM48A |
| FAS |
| FKBP1A |
| FKBP1B |
| FOS |
| FOXO1 |
| FOXO3 |
| GAA |
| GAA |
| GABARAP |
| GABARAP |
| GABARAPL1 |
| GABARAPL1 |
| GABARAPL2 |
| GABARAPL2 |
| GAPDH |
| GAPDH |
| GNAI3 |
| GNAI3 |
| GNB2L1 |
| GNB2L1 |
| GOPC |
| GOPC |
| GRID1 |
| GRID1 |
| GRID2 |
| GRID2 |
| HDAC1 |
| HDAC6 |
| HGS |
| HIF1A |
| HSP90AB1 |
| HSPA5 |
| HSPA8 |
| HSPB8 |
| IFNG |
| IKBKB |
| IKBKE |
| IL24 |
| IRGM |
| ITGA3 |
| ITGA6 |
| ITGB1 |
| ITGB4 |
| ITPR1 |
| KIAA0226 |
| KIAA0652 |
| KIAA0831 |
| KIF5B |
| KLHL24 |
| LAMP1 |
| LAMP2 |
| MAP1LC3A |
| MAP1LC3B |
| MAP1LC3C |
| MAP2K7 |
| MAPK1 |
| MAPK3 |
| MAPK8 |
| MAPK8IP1 |
| MAPK9 |
| MBTPS2 |
| MLST8 |
| MTMR14 |
| MTOR |
| MYC |
| NAF1 |
| NAMPT |
| NBR1 |
| NCKAP1 |
| NFE2L2 |
| NFKB1 |
| NKX2-3 |
| NLRC4 |
| NPC1 |
| NRG1 |
| NRG2 |
| NRG3 |
| P4HB |
| PARK2 |
| PARP1 |
| PEA15 |
| PELP1 |
| PEX14 |
| PEX3 |
| PIK3C3 |
| PIK3R4 |
| PINK1 |
| PPP1R15A |
| PRKAB1 |
| PRKAR1A |
| PRKCD |
| PRKCQ |
| PTEN |
| PTK6 |
| RAB11A |
| RAB1A |
| RAB24 |
| RAB33B |
| RAB5A |
| RAB7A |
| RAC1 |
| RAF1 |
| RB1 |
| RB1CC1 |
| RELA |
| RGS19 |
| RHEB |
| RPS6KB1 |
| RPTOR |
| SAR1A |
| SERPINA1 |
| SESN2 |
| SH3GLB1 |
| SIRT1 |
| SIRT2 |
| SPHK1 |
| SPNS1 |
| SQSTM1 |
| ST13 |
| STK11 |
| TBK1 |
| TM9SF1 |
| TMEM49 |
| TMEM74 |
| TNFSF10 |
| TP53 |
| TP53INP2 |
| TP63 |
| TP73 |
| TSC1 |
| TSC2 |
| TUSC1 |
| ULK1 |
| ULK2 |
| ULK3 |
| USP10 |
| UVRAG |
| VAMP3 |
| VAMP7 |
| VEGFA |
| WDFY3 |
| WDR45 |
| WDR45L |
| WIPI1 |
| WIPI2 |
| ZFYVE1 |
