# Supplementary material for: Mesenchymal Stem Cells May Alleviate the Intervertebral Disc Degeneration by Reducing the Oxidative Stress in Nucleus Pulposus Cells
Source: Stem Cells Int. 2022 Oct 3;2022:6082377. doi: 10.1155/2022/6082377 (PMC9551678; doi:10.1155/2022/6082377)
Supplement: Supplementary 3 — Supplementary Table 3. Detailed information of ceRNA regulatory network. [file 6082377.f3.docx]

**Supplementary Table 3. Detailed information of ceRNA regulatory network.**

| **DElncRNAs** | **miRNAs** | **hub OSRDEGs** |
| --- | --- | --- |
| ASAP1-IT2 | hsa-miR-185-5p | PTGS2 |
| ASAP1-IT2 | hsa-miR-4644 | PTGS2 |
| DLEU2 | hsa-miR-6509-3p | JUN |
| DLEU2 | hsa-miR-522-3p | JUN |
| DLEU2 | hsa-miR-224-3p | JUN |
| DLEU2 | hsa-miR-655-3p | JUN |
| DLEU2 | hsa-miR-374c-5p | JUN |
| DLEU2 | hsa-miR-4428 | JUN |
| DLEU2 | hsa-miR-212-5p | JUN |
| DNM3OS | hsa-miR-126-5p | CAT |
| GAS5 | hsa-miR-524-5p | CAT |
| GAS5 | hsa-miR-520d-5p | CAT |
| GAS5 | hsa-miR-361-5p | CAT |
| GAS5 | hsa-miR-5094 | CAT |
| GAS5 | hsa-miR-374a-5p | CAT |
| GAS5 | hsa-miR-374b-5p | CAT |
| GAS5 | hsa-miR-664b-3p | CAT |
| GAS5 | hsa-miR-579-3p | CAT |
| GAS5 | hsa-miR-369-3p | CAT |
| GAS5 | hsa-miR-548o-3p | CAT |
| GAS5 | hsa-miR-1323 | CAT |
| GAS5 | hsa-miR-205-5p | CAT |
| GAS5 | hsa-miR-576-5p | CAT |
| LINC01133 | hsa-miR-495-3p | EDN1 |
| LINC01133 | hsa-miR-5688 | EDN1 |
| LINC01133 | hsa-miR-625-5p | EDN1 |
| LINC01133 | hsa-miR-7151-5p | FOS |
| LINC01133 | hsa-miR-4640-5p | FOS |
| LINC01133 | hsa-miR-4726-5p | FOS |
| LINC01133 | hsa-miR-495-3p | JUN |
| LINC01133 | hsa-miR-5688 | JUN |
| LINC01133 | hsa-miR-625-5p | JUN |
| LINC01133 | hsa-miR-495-3p | KLF2 |
| LINC01133 | hsa-miR-5688 | KLF2 |
| LINC01133 | hsa-miR-625-5p | KLF2 |
| LINC01133 | hsa-miR-199a-5p | KLF2 |
| LINC01133 | hsa-miR-199b-5p | KLF2 |
| LINC01133 | hsa-miR-495-3p | PTGS2 |
| LINC01133 | hsa-miR-5688 | PTGS2 |
| LINC01133 | hsa-miR-7151-5p | PTGS2 |
| LINC01133 | hsa-miR-495-3p | TLR4 |
| LINC01133 | hsa-miR-5688 | TLR4 |
| LINC01133 | hsa-miR-4784 | TLR4 |
| LINC01133 | hsa-miR-3150b-3p | TLR4 |
| LINC01133 | hsa-miR-7151-5p | TLR4 |
| LINC01133 | hsa-miR-495-3p | TXNRD1 |
| LINC01133 | hsa-miR-5688 | TXNRD1 |
| LINC01133 | hsa-miR-7151-5p | TXNRD1 |
| LINC01133 | hsa-miR-625-5p | TXNRD1 |
| LINC01133 | hsa-miR-199a-5p | TXNRD1 |
| LINC01133 | hsa-miR-199b-5p | TXNRD1 |
| LINC01139 | hsa-miR-30a-5p | CAT |
| LINC01139 | hsa-miR-30d-5p | CAT |
| LINC01139 | hsa-miR-30b-5p | CAT |
| LINC01139 | hsa-miR-30e-5p | CAT |
| LINC01139 | hsa-miR-30c-5p | CAT |
| LINC02035 | hsa-miR-130a-5p | LRRK2 |
| LINC02035 | hsa-miR-23a-3p | LRRK2 |
| LINC02035 | hsa-miR-23b-3p | LRRK2 |
| LINC02035 | hsa-miR-23c | LRRK2 |
| LINC02035 | hsa-miR-5581-3p | LRRK2 |
| LINC02035 | hsa-miR-1252-5p | LRRK2 |
| LINC02035 | hsa-miR-519c-5p | LRRK2 |
| LINC02035 | hsa-miR-518f-5p | LRRK2 |
| LINC02035 | hsa-miR-526a | LRRK2 |
| LINC02035 | hsa-miR-523-5p | LRRK2 |
| LINC02035 | hsa-miR-518e-5p | LRRK2 |
| LINC02035 | hsa-miR-522-5p | LRRK2 |
| LINC02035 | hsa-miR-519a-5p | LRRK2 |
| LINC02035 | hsa-miR-519b-5p | LRRK2 |
| LINC02035 | hsa-miR-520c-5p | LRRK2 |
| LINC02035 | hsa-miR-518d-5p | LRRK2 |
| LINC02035 | hsa-miR-142-5p | LRRK2 |
| LINC02035 | hsa-miR-5590-3p | LRRK2 |
| LINC02035 | hsa-miR-329-3p | LRRK2 |
| LINC02035 | hsa-miR-362-3p | LRRK2 |
| LINC02035 | hsa-miR-9-3p | LRRK2 |
| LINC02035 | hsa-miR-103a-3p | LRRK2 |
| LINC02035 | hsa-miR-107 | LRRK2 |
| LINC02035 | hsa-miR-323a-3p | LRRK2 |
| LINC02035 | hsa-miR-130a-5p | TXNRD1 |
| LINC02035 | hsa-miR-23a-3p | TXNRD1 |
| LINC02035 | hsa-miR-23b-3p | TXNRD1 |
| LINC02035 | hsa-miR-23c | TXNRD1 |
| LINC02035 | hsa-miR-656-3p | TXNRD1 |
| LINC02035 | hsa-miR-5581-3p | TXNRD1 |
| LINC02035 | hsa-miR-3163 | TXNRD1 |
| LINC02035 | hsa-miR-432-5p | TXNRD1 |
| LINC02035 | hsa-miR-512-3p | TXNRD1 |
| LINC02035 | hsa-miR-5691 | TXNRD1 |
| LINC02035 | hsa-miR-6805-3p | TXNRD1 |
| LINC02035 | hsa-miR-9-3p | TXNRD1 |
| LINC02035 | hsa-miR-330-3p | TXNRD1 |
| LINC02035 | hsa-miR-182-5p | TXNRD1 |
| LINC02381 | hsa-miR-506-5p | LRRK2 |
| LINC02381 | hsa-miR-2355-3p | LRRK2 |
| LINC02381 | hsa-miR-27b-3p | LRRK2 |
| LINC02381 | hsa-miR-27a-3p | LRRK2 |
| LINC02381 | hsa-miR-491-5p | TXNRD1 |
| LINC02381 | hsa-miR-3194-3p | TXNRD1 |
| LINC02381 | hsa-miR-552-3p | TXNRD1 |
| LINC02381 | hsa-miR-495-3p | TXNRD1 |
| LINC02381 | hsa-miR-5688 | TXNRD1 |
| LPP-AS2 | hsa-miR-411-3p | CAT |
| LPP-AS2 | hsa-miR-379-3p | CAT |
| MEG8 | hsa-let-7g-5p | EDN1 |
| MEG8 | hsa-let-7i-5p | EDN1 |
| MEG8 | hsa-let-7a-5p | EDN1 |
| MEG8 | hsa-let-7b-5p | EDN1 |
| MEG8 | hsa-let-7c-5p | EDN1 |
| MEG8 | hsa-let-7d-5p | EDN1 |
| MEG8 | hsa-let-7e-5p | EDN1 |
| MEG8 | hsa-let-7f-5p | EDN1 |
| MEG8 | hsa-miR-98-5p | EDN1 |
| MEG8 | hsa-miR-4458 | EDN1 |
| MEG8 | hsa-miR-4500 | EDN1 |
| MEG8 | hsa-miR-650 | EDN1 |
| MEG8 | hsa-miR-3612 | EDN1 |
| MEG8 | hsa-miR-5688 | EDN1 |
| MEG8 | hsa-miR-495-3p | EDN1 |
| MEG8 | hsa-miR-3163 | EDN1 |
| MEG8 | hsa-miR-367-3p | JUN |
| MEG8 | hsa-miR-363-3p | JUN |
| MEG8 | hsa-miR-25-3p | JUN |
| MEG8 | hsa-miR-32-5p | JUN |
| MEG8 | hsa-miR-92a-3p | JUN |
| MEG8 | hsa-miR-92b-3p | JUN |
| MEG8 | hsa-miR-5688 | JUN |
| MEG8 | hsa-miR-495-3p | JUN |
| MEG8 | hsa-miR-16-5p | KLF2 |
| MEG8 | hsa-miR-6838-5p | KLF2 |
| MEG8 | hsa-miR-424-5p | KLF2 |
| MEG8 | hsa-miR-15a-5p | KLF2 |
| MEG8 | hsa-miR-15b-5p | KLF2 |
| MEG8 | hsa-miR-195-5p | KLF2 |
| MEG8 | hsa-miR-497-5p | KLF2 |
| MEG8 | hsa-miR-3194-5p | KLF2 |
| MEG8 | hsa-let-7g-5p | KLF2 |
| MEG8 | hsa-let-7i-5p | KLF2 |
| MEG8 | hsa-let-7a-5p | KLF2 |
| MEG8 | hsa-let-7b-5p | KLF2 |
| MEG8 | hsa-let-7c-5p | KLF2 |
| MEG8 | hsa-let-7d-5p | KLF2 |
| MEG8 | hsa-let-7e-5p | KLF2 |
| MEG8 | hsa-let-7f-5p | KLF2 |
| MEG8 | hsa-miR-98-5p | KLF2 |
| MEG8 | hsa-miR-4458 | KLF2 |
| MEG8 | hsa-miR-4500 | KLF2 |
| MEG8 | hsa-miR-1179 | KLF2 |
| MEG8 | hsa-miR-181a-5p | KLF2 |
| MEG8 | hsa-miR-181b-5p | KLF2 |
| MEG8 | hsa-miR-181c-5p | KLF2 |
| MEG8 | hsa-miR-181d-5p | KLF2 |
| MEG8 | hsa-miR-4262 | KLF2 |
| MEG8 | hsa-miR-367-3p | KLF2 |
| MEG8 | hsa-miR-3681-3p | KLF2 |
| MEG8 | hsa-miR-216a-3p | KLF2 |
| MEG8 | hsa-miR-128-3p | KLF2 |
| MEG8 | hsa-miR-363-3p | KLF2 |
| MEG8 | hsa-miR-25-3p | KLF2 |
| MEG8 | hsa-miR-32-5p | KLF2 |
| MEG8 | hsa-miR-92a-3p | KLF2 |
| MEG8 | hsa-miR-92b-3p | KLF2 |
| MEG8 | hsa-miR-616-3p | KLF2 |
| MEG8 | hsa-miR-3184-5p | KLF2 |
| MEG8 | hsa-miR-423-5p | KLF2 |
| MEG8 | hsa-miR-500a-3p | KLF2 |
| MEG8 | hsa-miR-545-3p | KLF2 |
| MEG8 | hsa-miR-650 | KLF2 |
| MEG8 | hsa-miR-3612 | KLF2 |
| MEG8 | hsa-miR-5688 | KLF2 |
| MEG8 | hsa-miR-495-3p | KLF2 |
| MEG8 | hsa-miR-3163 | KLF2 |
| MEG8 | hsa-miR-296-3p | KLF2 |
| MEG8 | hsa-miR-4761-5p | TLR4 |
| MEG8 | hsa-miR-16-5p | TLR4 |
| MEG8 | hsa-miR-6838-5p | TLR4 |
| MEG8 | hsa-miR-424-5p | TLR4 |
| MEG8 | hsa-miR-15a-5p | TLR4 |
| MEG8 | hsa-miR-15b-5p | TLR4 |
| MEG8 | hsa-miR-195-5p | TLR4 |
| MEG8 | hsa-miR-497-5p | TLR4 |
| MEG8 | hsa-let-7g-5p | TLR4 |
| MEG8 | hsa-let-7i-5p | TLR4 |
| MEG8 | hsa-let-7a-5p | TLR4 |
| MEG8 | hsa-let-7b-5p | TLR4 |
| MEG8 | hsa-let-7c-5p | TLR4 |
| MEG8 | hsa-let-7d-5p | TLR4 |
| MEG8 | hsa-let-7e-5p | TLR4 |
| MEG8 | hsa-let-7f-5p | TLR4 |
| MEG8 | hsa-miR-98-5p | TLR4 |
| MEG8 | hsa-miR-4458 | TLR4 |
| MEG8 | hsa-miR-4500 | TLR4 |
| MEG8 | hsa-miR-1179 | TLR4 |
| MEG8 | hsa-miR-181a-5p | TLR4 |
| MEG8 | hsa-miR-181b-5p | TLR4 |
| MEG8 | hsa-miR-181c-5p | TLR4 |
| MEG8 | hsa-miR-181d-5p | TLR4 |
| MEG8 | hsa-miR-4262 | TLR4 |
| MEG8 | hsa-miR-367-3p | TLR4 |
| MEG8 | hsa-miR-363-3p | TLR4 |
| MEG8 | hsa-miR-25-3p | TLR4 |
| MEG8 | hsa-miR-32-5p | TLR4 |
| MEG8 | hsa-miR-92a-3p | TLR4 |
| MEG8 | hsa-miR-92b-3p | TLR4 |
| MEG8 | hsa-miR-582-5p | TLR4 |
| MEG8 | hsa-miR-31-5p | TLR4 |
| MEG8 | hsa-miR-3184-5p | TLR4 |
| MEG8 | hsa-miR-423-5p | TLR4 |
| MEG8 | hsa-miR-23a-3p | TLR4 |
| MEG8 | hsa-miR-23b-3p | TLR4 |
| MEG8 | hsa-miR-130a-5p | TLR4 |
| MEG8 | hsa-miR-23c | TLR4 |
| MEG8 | hsa-miR-885-5p | TLR4 |
| MEG8 | hsa-miR-103a-3p | TLR4 |
| MEG8 | hsa-miR-107 | TLR4 |
| MEG8 | hsa-miR-650 | TLR4 |
| MEG8 | hsa-miR-3612 | TLR4 |
| MEG8 | hsa-miR-485-3p | TLR4 |
| MEG8 | hsa-miR-539-3p | TLR4 |
| MEG8 | hsa-miR-5688 | TLR4 |
| MEG8 | hsa-miR-495-3p | TLR4 |
| MEG8 | hsa-miR-3163 | TLR4 |
| MEG8 | hsa-miR-296-3p | TLR4 |
| MIR100HG | hsa-miR-3163 | EDN1 |
| MIR100HG | hsa-miR-5590-3p | EDN1 |
| MIR100HG | hsa-miR-142-5p | EDN1 |
| MIR100HG | hsa-miR-2467-3p | EDN1 |
| MIR100HG | hsa-miR-6763-5p | EDN1 |
| MIR100HG | hsa-miR-3150a-3p | EDN1 |
| MIR100HG | hsa-miR-519e-5p | EDN1 |
| MIR100HG | hsa-miR-515-5p | EDN1 |
| MIR100HG | hsa-miR-3163 | FOS |
| MIR100HG | hsa-miR-5590-3p | FOS |
| MIR100HG | hsa-miR-142-5p | FOS |
| MIR100HG | hsa-miR-2355-3p | FOS |
| MIR100HG | hsa-miR-519e-5p | FOS |
| MIR100HG | hsa-miR-515-5p | FOS |
| MIR100HG | hsa-miR-27b-3p | TLR4 |
| MIR100HG | hsa-miR-27a-3p | TLR4 |
| MIR100HG | hsa-miR-3163 | TLR4 |
| MIR100HG | hsa-miR-146a-5p | TLR4 |
| MIR100HG | hsa-miR-146b-5p | TLR4 |
| MIR100HG | hsa-miR-7153-5p | TLR4 |
| MIR100HG | hsa-miR-5590-3p | TLR4 |
| MIR100HG | hsa-miR-142-5p | TLR4 |
| MIR100HG | hsa-miR-374b-3p | TLR4 |
| MIR100HG | hsa-miR-2355-3p | TLR4 |
| MIR100HG | hsa-miR-6763-5p | TLR4 |
| MIR100HG | hsa-miR-3150a-3p | TLR4 |
| MIR100HG | hsa-miR-625-3p | TLR4 |
| MIR100HG | hsa-miR-4766-5p | TLR4 |
| MIR100HG | hsa-miR-942-5p | TLR4 |
| MIR100HG | hsa-miR-519e-5p | TLR4 |
| MIR100HG | hsa-miR-515-5p | TLR4 |
| MIR100HG | hsa-miR-3163 | TXNRD1 |
| MIR100HG | hsa-miR-374b-3p | TXNRD1 |
| MIR100HG | hsa-miR-625-3p | TXNRD1 |
| MIR100HG | hsa-miR-488-3p | TXNRD1 |
| MIR4435-2HG | hsa-miR-2467-3p | EDN1 |
| MIR4435-2HG | hsa-miR-4525 | EDN1 |
| MIR4435-2HG | hsa-miR-5010-5p | EDN1 |
| MIR4435-2HG | hsa-miR-670-5p | EDN1 |
| MIR4435-2HG | hsa-miR-125b-5p | EDN1 |
| MIR4435-2HG | hsa-miR-125a-5p | EDN1 |
| MIR4435-2HG | hsa-miR-4319 | EDN1 |
| MIR4435-2HG | hsa-miR-613 | EDN1 |
| MIR4435-2HG | hsa-miR-1-3p | EDN1 |
| MIR4435-2HG | hsa-miR-206 | EDN1 |
| MIR4435-2HG | hsa-miR-802 | FOS |
| MIR4435-2HG | hsa-miR-520h | FOS |
| MIR4435-2HG | hsa-miR-520g-3p | FOS |
| MIR4435-2HG | hsa-miR-149-5p | FOS |
| MIR4435-2HG | hsa-miR-136-5p | FOS |
| MIR4435-2HG | hsa-miR-627-5p | FOS |
| MIR4435-2HG | hsa-miR-24-3p | FOS |
| MIR4435-2HG | hsa-miR-642a-5p | JUN |
| MIR4435-2HG | hsa-miR-212-5p | JUN |
| MIR4435-2HG | hsa-miR-149-5p | JUN |
| MIR4435-2HG | hsa-miR-4525 | JUN |
| MIR4435-2HG | hsa-miR-5010-5p | JUN |
| MIR4435-2HG | hsa-miR-6512-3p | JUN |
| MIR4435-2HG | hsa-miR-6720-5p | JUN |
| MIR4435-2HG | hsa-miR-24-3p | JUN |
| MIR4435-2HG | hsa-miR-330-5p | KLF2 |
| MIR4435-2HG | hsa-miR-378g | KLF2 |
| MIR4435-2HG | hsa-miR-374a-3p | KLF2 |
| MIR4435-2HG | hsa-miR-520h | KLF2 |
| MIR4435-2HG | hsa-miR-520g-3p | KLF2 |
| MIR4435-2HG | hsa-miR-371a-5p | KLF2 |
| MIR4435-2HG | hsa-miR-212-5p | KLF2 |
| MIR4435-2HG | hsa-miR-500a-3p | KLF2 |
| MIR4435-2HG | hsa-miR-873-3p | KLF2 |
| MIR4435-2HG | hsa-miR-665 | KLF2 |
| MIR4435-2HG | hsa-miR-627-5p | KLF2 |
| MIR4435-2HG | hsa-miR-1343-3p | KLF2 |
| MIR4435-2HG | hsa-miR-6783-3p | KLF2 |
| MIR4435-2HG | hsa-miR-3187-3p | KLF2 |
| MIR4435-2HG | hsa-miR-641 | KLF2 |
| MIR4435-2HG | hsa-miR-513a-5p | KLF2 |
| MIR4435-2HG | hsa-miR-7853-5p | KLF2 |
| MIR4435-2HG | hsa-miR-105-5p | KLF2 |
| MIR4435-2HG | hsa-miR-2467-3p | KLF2 |
| MIR4435-2HG | hsa-miR-128-3p | KLF2 |
| MIR4435-2HG | hsa-miR-3681-3p | KLF2 |
| MIR4435-2HG | hsa-miR-216a-3p | KLF2 |
| MIR4435-2HG | hsa-miR-4525 | KLF2 |
| MIR4435-2HG | hsa-miR-5010-5p | KLF2 |
| MIR4435-2HG | hsa-miR-370-5p | KLF2 |
| MIR4435-2HG | hsa-miR-6512-3p | KLF2 |
| MIR4435-2HG | hsa-miR-6720-5p | KLF2 |
| MIR4435-2HG | hsa-miR-125b-5p | KLF2 |
| MIR4435-2HG | hsa-miR-125a-5p | KLF2 |
| MIR4435-2HG | hsa-miR-4319 | KLF2 |
| MIR4435-2HG | hsa-miR-485-5p | KLF2 |
| MIR4435-2HG | hsa-miR-6884-5p | KLF2 |
| MIR4435-2HG | hsa-miR-3622a-5p | KLF2 |
| MIR4435-2HG | hsa-miR-24-3p | KLF2 |
| MIR4435-2HG | hsa-miR-503-5p | KLF2 |
| MIR4435-2HG | hsa-miR-1252-5p | KLF2 |
| MIR4435-2HG | hsa-miR-205-5p | KLF2 |
| MIR4435-2HG | hsa-miR-330-5p | TLR4 |
| MIR4435-2HG | hsa-miR-378g | TLR4 |
| MIR4435-2HG | hsa-miR-374a-3p | TLR4 |
| MIR4435-2HG | hsa-miR-802 | TLR4 |
| MIR4435-2HG | hsa-miR-520h | TLR4 |
| MIR4435-2HG | hsa-miR-520g-3p | TLR4 |
| MIR4435-2HG | hsa-miR-371a-5p | TLR4 |
| MIR4435-2HG | hsa-miR-642a-5p | TLR4 |
| MIR4435-2HG | hsa-miR-212-5p | TLR4 |
| MIR4435-2HG | hsa-miR-149-5p | TLR4 |
| MIR4435-2HG | hsa-miR-2116-3p | TLR4 |
| MIR4435-2HG | hsa-miR-665 | TLR4 |
| MIR4435-2HG | hsa-miR-1224-5p | TLR4 |
| MIR4435-2HG | hsa-miR-136-5p | TLR4 |
| MIR4435-2HG | hsa-miR-627-5p | TLR4 |
| MIR4435-2HG | hsa-miR-1343-3p | TLR4 |
| MIR4435-2HG | hsa-miR-6783-3p | TLR4 |
| MIR4435-2HG | hsa-miR-641 | TLR4 |
| MIR4435-2HG | hsa-miR-582-5p | TLR4 |
| MIR4435-2HG | hsa-miR-513a-5p | TLR4 |
| MIR4435-2HG | hsa-miR-7853-5p | TLR4 |
| MIR4435-2HG | hsa-miR-105-5p | TLR4 |
| MIR4435-2HG | hsa-miR-376a-3p | TLR4 |
| MIR4435-2HG | hsa-miR-376b-3p | TLR4 |
| MIR4435-2HG | hsa-miR-370-5p | TLR4 |
| MIR4435-2HG | hsa-miR-670-5p | TLR4 |
| MIR4435-2HG | hsa-miR-125b-5p | TLR4 |
| MIR4435-2HG | hsa-miR-125a-5p | TLR4 |
| MIR4435-2HG | hsa-miR-4319 | TLR4 |
| MIR4435-2HG | hsa-miR-485-5p | TLR4 |
| MIR4435-2HG | hsa-miR-6884-5p | TLR4 |
| MIR4435-2HG | hsa-miR-3611 | TLR4 |
| MIR4435-2HG | hsa-miR-24-3p | TLR4 |
| MIR4435-2HG | hsa-miR-613 | TLR4 |
| MIR4435-2HG | hsa-miR-1-3p | TLR4 |
| MIR4435-2HG | hsa-miR-206 | TLR4 |
| MIR4435-2HG | hsa-miR-503-5p | TLR4 |
| MIR4435-2HG | hsa-miR-1252-5p | TLR4 |
| MIR4435-2HG | hsa-miR-205-5p | TLR4 |
| MIR4435-2HG | hsa-miR-802 | TXNRD1 |
| MIR4435-2HG | hsa-miR-873-3p | TXNRD1 |
| MIR4435-2HG | hsa-miR-1224-5p | TXNRD1 |
| MIR4435-2HG | hsa-miR-7853-5p | TXNRD1 |
| MIR4435-2HG | hsa-miR-105-5p | TXNRD1 |
| MIR4435-2HG | hsa-miR-4525 | TXNRD1 |
| MIR4435-2HG | hsa-miR-5010-5p | TXNRD1 |
| MIR4435-2HG | hsa-miR-6512-3p | TXNRD1 |
| MIR4435-2HG | hsa-miR-6720-5p | TXNRD1 |
| MIR4435-2HG | hsa-miR-670-5p | TXNRD1 |
| MIR4435-2HG | hsa-miR-125b-5p | TXNRD1 |
| MIR4435-2HG | hsa-miR-125a-5p | TXNRD1 |
| MIR4435-2HG | hsa-miR-4319 | TXNRD1 |
| MIR4435-2HG | hsa-miR-3611 | TXNRD1 |
| MIR4435-2HG | hsa-miR-205-5p | TXNRD1 |
| N4BP2L2-IT2 | hsa-miR-4319 | EDN1 |
| N4BP2L2-IT2 | hsa-miR-125b-5p | EDN1 |
| N4BP2L2-IT2 | hsa-miR-125a-5p | EDN1 |
| N4BP2L2-IT2 | hsa-miR-3179 | EDN1 |
| N4BP2L2-IT2 | hsa-miR-340-5p | EDN1 |
| N4BP2L2-IT2 | hsa-miR-147a | EDN1 |
| N4BP2L2-IT2 | hsa-miR-376c-3p | EDN1 |
| N4BP2L2-IT2 | hsa-miR-561-5p | EDN1 |
| N4BP2L2-IT2 | hsa-miR-671-5p | EDN1 |
| N4BP2L2-IT2 | hsa-miR-656-3p | EDN1 |
| N4BP2L2-IT2 | hsa-miR-3605-5p | EDN1 |
| N4BP2L2-IT2 | hsa-miR-1294 | EDN1 |
| N4BP2L2-IT2 | hsa-miR-2355-5p | EDN1 |
| N4BP2L2-IT2 | hsa-miR-216a-5p | FOS |
| N4BP2L2-IT2 | hsa-miR-338-3p | FOS |
| N4BP2L2-IT2 | hsa-miR-543 | FOS |
| N4BP2L2-IT2 | hsa-miR-2115-3p | FOS |
| N4BP2L2-IT2 | hsa-miR-323b-3p | FOS |
| N4BP2L2-IT2 | hsa-miR-4761-5p | FOS |
| N4BP2L2-IT2 | hsa-miR-139-5p | FOS |
| N4BP2L2-IT2 | hsa-miR-191-5p | FOS |
| N4BP2L2-IT2 | hsa-miR-4262 | FOS |
| N4BP2L2-IT2 | hsa-miR-181c-5p | FOS |
| N4BP2L2-IT2 | hsa-miR-181a-5p | FOS |
| N4BP2L2-IT2 | hsa-miR-181b-5p | FOS |
| N4BP2L2-IT2 | hsa-miR-181d-5p | FOS |
| N4BP2L2-IT2 | hsa-miR-204-5p | FOS |
| N4BP2L2-IT2 | hsa-miR-211-5p | FOS |
| N4BP2L2-IT2 | hsa-miR-889-3p | FOS |
| N4BP2L2-IT2 | hsa-miR-7151-5p | FOS |
| N4BP2L2-IT2 | hsa-miR-365a-3p | FOS |
| N4BP2L2-IT2 | hsa-miR-365b-3p | FOS |
| N4BP2L2-IT2 | hsa-miR-2355-5p | FOS |
| N4BP2L2-IT2 | hsa-miR-543 | JUN |
| N4BP2L2-IT2 | hsa-miR-340-5p | JUN |
| N4BP2L2-IT2 | hsa-miR-323b-3p | JUN |
| N4BP2L2-IT2 | hsa-miR-342-3p | JUN |
| N4BP2L2-IT2 | hsa-miR-139-5p | JUN |
| N4BP2L2-IT2 | hsa-miR-493-5p | JUN |
| N4BP2L2-IT2 | hsa-miR-516b-5p | JUN |
| N4BP2L2-IT2 | hsa-miR-4319 | KLF2 |
| N4BP2L2-IT2 | hsa-miR-125b-5p | KLF2 |
| N4BP2L2-IT2 | hsa-miR-125a-5p | KLF2 |
| N4BP2L2-IT2 | hsa-miR-338-3p | KLF2 |
| N4BP2L2-IT2 | hsa-miR-543 | KLF2 |
| N4BP2L2-IT2 | hsa-miR-2115-3p | KLF2 |
| N4BP2L2-IT2 | hsa-miR-3179 | KLF2 |
| N4BP2L2-IT2 | hsa-miR-340-5p | KLF2 |
| N4BP2L2-IT2 | hsa-miR-323b-3p | KLF2 |
| N4BP2L2-IT2 | hsa-miR-342-3p | KLF2 |
| N4BP2L2-IT2 | hsa-miR-147a | KLF2 |
| N4BP2L2-IT2 | hsa-miR-491-5p | KLF2 |
| N4BP2L2-IT2 | hsa-miR-4262 | KLF2 |
| N4BP2L2-IT2 | hsa-miR-181c-5p | KLF2 |
| N4BP2L2-IT2 | hsa-miR-181a-5p | KLF2 |
| N4BP2L2-IT2 | hsa-miR-181b-5p | KLF2 |
| N4BP2L2-IT2 | hsa-miR-181d-5p | KLF2 |
| N4BP2L2-IT2 | hsa-miR-561-5p | KLF2 |
| N4BP2L2-IT2 | hsa-miR-374a-3p | KLF2 |
| N4BP2L2-IT2 | hsa-miR-3127-5p | KLF2 |
| N4BP2L2-IT2 | hsa-miR-488-3p | KLF2 |
| N4BP2L2-IT2 | hsa-miR-4735-3p | KLF2 |
| N4BP2L2-IT2 | hsa-miR-18a-5p | KLF2 |
| N4BP2L2-IT2 | hsa-miR-18b-5p | KLF2 |
| N4BP2L2-IT2 | hsa-miR-889-3p | KLF2 |
| N4BP2L2-IT2 | hsa-miR-506-5p | KLF2 |
| N4BP2L2-IT2 | hsa-miR-2278 | KLF2 |
| N4BP2L2-IT2 | hsa-miR-365a-3p | KLF2 |
| N4BP2L2-IT2 | hsa-miR-365b-3p | KLF2 |
| N4BP2L2-IT2 | hsa-miR-516b-5p | KLF2 |
| N4BP2L2-IT2 | hsa-miR-769-5p | KLF2 |
| N4BP2L2-IT2 | hsa-miR-877-5p | KLF2 |
| N4BP2L2-IT2 | hsa-miR-656-3p | KLF2 |
| N4BP2L2-IT2 | hsa-miR-361-5p | KLF2 |
| N4BP2L2-IT2 | hsa-miR-1294 | KLF2 |
| N4BP2L2-IT2 | hsa-miR-760 | KLF2 |
| N4BP2L2-IT2 | hsa-miR-942-5p | KLF2 |
| N4BP2L2-IT2 | hsa-miR-2355-5p | KLF2 |
| N4BP2L2-IT2 | hsa-miR-216a-5p | TLR4 |
| N4BP2L2-IT2 | hsa-miR-4319 | TLR4 |
| N4BP2L2-IT2 | hsa-miR-125b-5p | TLR4 |
| N4BP2L2-IT2 | hsa-miR-125a-5p | TLR4 |
| N4BP2L2-IT2 | hsa-miR-338-3p | TLR4 |
| N4BP2L2-IT2 | hsa-miR-543 | TLR4 |
| N4BP2L2-IT2 | hsa-miR-2115-3p | TLR4 |
| N4BP2L2-IT2 | hsa-miR-3179 | TLR4 |
| N4BP2L2-IT2 | hsa-miR-340-5p | TLR4 |
| N4BP2L2-IT2 | hsa-miR-660-5p | TLR4 |
| N4BP2L2-IT2 | hsa-miR-6509-5p | TLR4 |
| N4BP2L2-IT2 | hsa-miR-4761-5p | TLR4 |
| N4BP2L2-IT2 | hsa-miR-147a | TLR4 |
| N4BP2L2-IT2 | hsa-miR-491-5p | TLR4 |
| N4BP2L2-IT2 | hsa-miR-3171 | TLR4 |
| N4BP2L2-IT2 | hsa-miR-511-3p | TLR4 |
| N4BP2L2-IT2 | hsa-miR-493-5p | TLR4 |
| N4BP2L2-IT2 | hsa-miR-376c-3p | TLR4 |
| N4BP2L2-IT2 | hsa-miR-4262 | TLR4 |
| N4BP2L2-IT2 | hsa-miR-181c-5p | TLR4 |
| N4BP2L2-IT2 | hsa-miR-181a-5p | TLR4 |
| N4BP2L2-IT2 | hsa-miR-181b-5p | TLR4 |
| N4BP2L2-IT2 | hsa-miR-181d-5p | TLR4 |
| N4BP2L2-IT2 | hsa-miR-561-5p | TLR4 |
| N4BP2L2-IT2 | hsa-miR-374a-3p | TLR4 |
| N4BP2L2-IT2 | hsa-miR-4766-5p | TLR4 |
| N4BP2L2-IT2 | hsa-miR-23a-3p | TLR4 |
| N4BP2L2-IT2 | hsa-miR-23b-3p | TLR4 |
| N4BP2L2-IT2 | hsa-miR-130a-5p | TLR4 |
| N4BP2L2-IT2 | hsa-miR-23c | TLR4 |
| N4BP2L2-IT2 | hsa-miR-708-5p | TLR4 |
| N4BP2L2-IT2 | hsa-miR-3139 | TLR4 |
| N4BP2L2-IT2 | hsa-miR-28-5p | TLR4 |
| N4BP2L2-IT2 | hsa-miR-4735-3p | TLR4 |
| N4BP2L2-IT2 | hsa-miR-18a-5p | TLR4 |
| N4BP2L2-IT2 | hsa-miR-18b-5p | TLR4 |
| N4BP2L2-IT2 | hsa-miR-889-3p | TLR4 |
| N4BP2L2-IT2 | hsa-miR-7151-5p | TLR4 |
| N4BP2L2-IT2 | hsa-miR-671-5p | TLR4 |
| N4BP2L2-IT2 | hsa-miR-2278 | TLR4 |
| N4BP2L2-IT2 | hsa-miR-365a-3p | TLR4 |
| N4BP2L2-IT2 | hsa-miR-365b-3p | TLR4 |
| N4BP2L2-IT2 | hsa-miR-516b-5p | TLR4 |
| N4BP2L2-IT2 | hsa-miR-769-5p | TLR4 |
| N4BP2L2-IT2 | hsa-miR-877-5p | TLR4 |
| N4BP2L2-IT2 | hsa-miR-656-3p | TLR4 |
| N4BP2L2-IT2 | hsa-miR-361-5p | TLR4 |
| N4BP2L2-IT2 | hsa-miR-1294 | TLR4 |
| N4BP2L2-IT2 | hsa-miR-760 | TLR4 |
| N4BP2L2-IT2 | hsa-miR-942-5p | TLR4 |
| N4BP2L2-IT2 | hsa-miR-2355-5p | TLR4 |
| N4BP2L2-IT2 | hsa-miR-4319 | TXNRD1 |
| N4BP2L2-IT2 | hsa-miR-125b-5p | TXNRD1 |
| N4BP2L2-IT2 | hsa-miR-125a-5p | TXNRD1 |
| N4BP2L2-IT2 | hsa-miR-543 | TXNRD1 |
| N4BP2L2-IT2 | hsa-miR-2115-3p | TXNRD1 |
| N4BP2L2-IT2 | hsa-miR-491-5p | TXNRD1 |
| N4BP2L2-IT2 | hsa-miR-376c-3p | TXNRD1 |
| N4BP2L2-IT2 | hsa-miR-488-3p | TXNRD1 |
| N4BP2L2-IT2 | hsa-miR-23a-3p | TXNRD1 |
| N4BP2L2-IT2 | hsa-miR-23b-3p | TXNRD1 |
| N4BP2L2-IT2 | hsa-miR-130a-5p | TXNRD1 |
| N4BP2L2-IT2 | hsa-miR-23c | TXNRD1 |
| N4BP2L2-IT2 | hsa-miR-204-5p | TXNRD1 |
| N4BP2L2-IT2 | hsa-miR-211-5p | TXNRD1 |
| N4BP2L2-IT2 | hsa-miR-889-3p | TXNRD1 |
| N4BP2L2-IT2 | hsa-miR-7151-5p | TXNRD1 |
| N4BP2L2-IT2 | hsa-miR-365a-3p | TXNRD1 |
| N4BP2L2-IT2 | hsa-miR-365b-3p | TXNRD1 |
| N4BP2L2-IT2 | hsa-miR-516b-5p | TXNRD1 |
| N4BP2L2-IT2 | hsa-miR-656-3p | TXNRD1 |
| N4BP2L2-IT2 | hsa-miR-361-5p | TXNRD1 |
| N4BP2L2-IT2 | hsa-miR-3605-5p | TXNRD1 |
| N4BP2L2-IT2 | hsa-miR-760 | TXNRD1 |
| PSMG3-AS1 | hsa-miR-885-5p | LRRK2 |
| PSMG3-AS1 | hsa-miR-384 | LRRK2 |
| PSMG3-AS1 | hsa-miR-5009-3p | LRRK2 |
| PSMG3-AS1 | hsa-miR-181b-5p | LRRK2 |
| PSMG3-AS1 | hsa-miR-181d-5p | LRRK2 |
| PSMG3-AS1 | hsa-miR-181a-5p | LRRK2 |
| PSMG3-AS1 | hsa-miR-181c-5p | LRRK2 |
| PSMG3-AS1 | hsa-miR-4262 | LRRK2 |
| PSMG3-AS1 | hsa-miR-1224-5p | LRRK2 |
| SBF2-AS1 | hsa-miR-3612 | EDN1 |
| SBF2-AS1 | hsa-miR-650 | EDN1 |
| SBF2-AS1 | hsa-miR-345-3p | EDN1 |
| SBF2-AS1 | hsa-miR-670-5p | EDN1 |
| SBF2-AS1 | hsa-miR-338-3p | TLR4 |
| SBF2-AS1 | hsa-miR-329-3p | TLR4 |
| SBF2-AS1 | hsa-miR-362-3p | TLR4 |
| SBF2-AS1 | hsa-miR-3612 | TLR4 |
| SBF2-AS1 | hsa-miR-650 | TLR4 |
| SBF2-AS1 | hsa-miR-1252-5p | TLR4 |
| SBF2-AS1 | hsa-miR-151a-3p | TLR4 |
| SBF2-AS1 | hsa-miR-582-5p | TLR4 |
| SBF2-AS1 | hsa-miR-140-5p | TLR4 |
| SBF2-AS1 | hsa-miR-520f-3p | TLR4 |
| SBF2-AS1 | hsa-miR-302d-3p | TLR4 |
| SBF2-AS1 | hsa-miR-372-3p | TLR4 |
| SBF2-AS1 | hsa-miR-373-3p | TLR4 |
| SBF2-AS1 | hsa-miR-520a-3p | TLR4 |
| SBF2-AS1 | hsa-miR-520c-3p | TLR4 |
| SBF2-AS1 | hsa-miR-520d-3p | TLR4 |
| SBF2-AS1 | hsa-miR-520b | TLR4 |
| SBF2-AS1 | hsa-miR-302a-3p | TLR4 |
| SBF2-AS1 | hsa-miR-302b-3p | TLR4 |
| SBF2-AS1 | hsa-miR-520e | TLR4 |
| SBF2-AS1 | hsa-miR-302e | TLR4 |
| SBF2-AS1 | hsa-miR-2115-3p | TLR4 |
| SBF2-AS1 | hsa-miR-4766-5p | TLR4 |
| SBF2-AS1 | hsa-miR-345-3p | TLR4 |
| SBF2-AS1 | hsa-miR-670-5p | TLR4 |
| SBF2-AS1 | hsa-miR-541-5p | TLR4 |
| SNHG8 | hsa-miR-425-5p | CAT |
| SNHG8 | hsa-miR-384 | CAT |
| ZNF561-AS1 | hsa-miR-185-5p | APOE |
| ZNF561-AS1 | hsa-miR-4644 | APOE |

ceRNA, competing endogenous RNA; DElncRNAs, differentially expressed long noncoding RNAs; OSRDEGs, oxidative stress related differentially expressed genes.
